# Primary Graft Dysfunction: The Role of Aging in Lung Ischemia-Reperfusion Injury

**DOI:** 10.3389/fimmu.2022.891564

**Published:** 2022-05-24

**Authors:** Maximilian J. Roesel, Nirmal S. Sharma, Andreas Schroeter, Tomohisa Matsunaga, Yao Xiao, Hao Zhou, Stefan G. Tullius

**Affiliations:** ^1^ Division of Transplant Surgery and Transplant Surgery Research Laboratory, Brigham and Women’s Hospital, Harvard Medical School, Boston, MA, United States; ^2^ Institute of Medical Immunology, Charité Universitaetsmedizin Berlin, Berlin, Germany; ^3^ Division of Pulmonary and Critical Care Medicine, Brigham and Women’s Hospital, Boston, MA, United States; ^4^ Department of Medicine, Harvard Medical School, Boston, MA, United States; ^5^ Regenerative Medicine and Experimental Surgery, Department of General, Visceral and Transplant Surgery, Hannover Medical School, Hannover, Germany; ^6^ Department of Urology, Osaka Medical and Pharmaceutical University, Osaka, Japan

**Keywords:** aging, ischemia reperfusion injury, primary graft dysfunction, lung transplantation, senolytics

## Abstract

Transplant centers around the world have been using extended criteria donors to remedy the ongoing demand for lung transplantation. With a rapidly aging population, older donors are increasingly considered. Donor age, at the same time has been linked to higher rates of lung ischemia reperfusion injury (IRI). This process of acute, sterile inflammation occurring upon reperfusion is a key driver of primary graft dysfunction (PGD) leading to inferior short- and long-term survival. Understanding and improving the condition of older lungs is thus critical to optimize outcomes. Notably, *ex vivo* lung perfusion (EVLP) seems to have the potential of reconditioning ischemic lungs through *ex-vivo* perfusing and ventilation. Here, we aim to delineate mechanisms driving lung IRI and review both experimental and clinical data on the effects of aging in augmenting the consequences of IRI and PGD in lung transplantation.

## Introduction

The world population is aging rapidly: predications suggest that more than 1.5 billion people above the age of 65 will inhabit our planet in 2050, accounting for 30 percent of the population (1). Similar trends are also observed in organ transplantation with increasing proportions of both, elderly donors and recipients (2, 3). Most donors are currently > than 50 years old and the proportion of donors > than 65 years has increased from 1 to 8% during the last decade (4, 5). United Network for Organ Sharing (UNOS) modeling suggests a potential of 22,000 available older donors/year (50-75 years) that are currently not considered (6). At least in theory, organs of those donors have the potential to narrow the gap between the ever-growing demand while measurements improving quality may therefore address prolonged waiting times and mortality on the waitlist. Not considering those organs or discarding them has been based on concerns of inferior outcomes (7, 8). Notably, it has been shown that older donor age decreases graft survival in hepatic, renal and heart transplantation (9–11). It is therefore likely that similar effects may be relevant in lung transplantation (12).

While lung donor selection criteria have historically been strict, thus limiting the donor pool, more recently, several transplant centers have used extended criteria donors including those from older donors with non-inferior outcomes (13–20). The clinical utilization of organs from donors > 65 years has nevertheless remained infrequent. A retrospective study examining outcomes of > than 10,000 lung transplant recipients reported an increase in 1- and 3-year mortality when transplanting lungs from donors >65 year. Using lungs from donors aged 55-64, however, has not been a risk factor for mortality and survival differences based on donor age have not been observed 30 days after transplantation (21). Primary graft dysfunction (PGD) represents one of the main risk factors for inferior short- and long-term survival (22–24). Interestingly, it remains unclear if donor age represents an independent risk factor for PGD (25–28).

## Clinical Impact of Primary Graft Dysfunction on Lung Transplant Outcomes

Primary graft dysfunction is characterized by hypoxemia and alveolar infiltrates in the allograft within the first 72 hours after lung transplantation (29–31). Transplant-mediated immune signals originating from endothelial, epithelial cells and alveolar macrophages lead to excessive infiltration of monocytes, neutrophils, and T-cells. The subsequent release of pro-inflammatory cytokines, reactive oxygen intermediates, and proteolytic enzymes lead to graft dysfunction (32). Based on the International Society for Heart and Lung Transplantation (ISHLT) standardized definition, the severity of the injury is graded by a PGD score between 0-3 score with PGD-3 being the most severe stage (**Table 1**). By using only two clinical parameters (radiographic infiltrates, PaO_2_/FiO_2_ ratio) the classification contains both a time- and severity component, facilitating mechanistic and clinical trials (24, 29, 33). An analysis of the UNOS database including 7,322 first-time lung transplant recipients reported a 72hr post-transplant PGD-2 and 3 rates of 8.2% and 20.8%, respectively, findings that are in line with previous reports by others (34, 35). The incidence of PGD rates may differ depending on the assessment after transplantation with PGD-3 incidences of 19.8% and 15.4% reported 48 and 72hrs after transplantation (26). Although the risk of advanced PGD (stage 3) seems to be declining by day 3 after transplantation, the occurrence and its severity has a significant negative impact on both, short- and long-term survival (36). An analysis of > than 5,000 lung transplant recipients of the UNOS database reported a 30 day post-lung transplantation mortality of 9.7%, among which 43.6% had PGD indicating its role in defining early post-transplant outcomes after lung transplantation (37). In an additional single center cohort study of 1,000 adult lung recipients, medium- and long-term survival rates were significantly compromised in those that experienced PGD (graft survival by 1-, 5-, and 10-years in recipients with and without PGD: 72.8 vs. 87.1% vs., 43.9 vs. 59.8%, and 18.7% vs. 35.7%, p<0.001) (36). PGD was also identified as a risk factor for the development of bronchiolitis obliterans syndrome and other forms of chronic lung allograft rejection (36, 38).

**Table 1:** ISHLT Primary Graft Dysfunction grading schema.

PGD Grade	Radiographic Infiltrates^1^	PaO_2_/FiO_2_
0	Absent	Any
1	Present	>300
2	Present	200-300
3	Present	<200
Time points^2^	T0: Within 6 hours of reperfusion	
	T24, T48, T72: 24, 48, 72 hours after reperfusion	

^1^Consistent with Pulmonary Edema, ^2^Use worse PaO_2_/FiO_2_ if multiple readings are available. Abbreviations: PGD, Primary Graft Dysfunction; PaO_2_, partial arterial pressure of oxygen; FiO_2_, fraction of inspired oxygen.

Based on the clinical significance, several studies have been conducted aiming to define risk factors (39–43). A systemic review and meta-analysis of 13 studies published between 2000 to 2013 identified female gender, African American race, idiopathic pulmonary fibrosis, sarcoidosis, primary pulmonary hypertension, elevated BMI, and the use of cardiopulmonary bypass as significant risk factors for development of PGD. However, donor age as a risk for PGD was not assessed in the meta-analysis (25). Additional risk factors include a history of cigarette smoking and single lung transplants; cut-off times for ischemia have been discussed controversially (44–47). In 2016, the ISHLT working group reviewed donor, recipient, and surgical risk factors and found an association between donor age and reduced long-term survival, but its impact on PGD remain was unclear (48). While an earlier study reported a 7-fold increase in risk of severe PGD with donors beyond the age of 45 years, more recent studies have failed to confirm this association (28, 42, 49). At the same time, ischemia reperfusion injury (IRI) has been recognized as a key driver of PGD supported by a very recent integrated bioinformatics analysis that identified various IRI-PGD common pathways (25, 32, 50). Thus, it is crucial to understand the mechanisms which drive IRI to improve outcomes after lung transplantation.

## Lung Ischemia Reperfusion Injury

Ischemia reperfusion injury represents an exacerbation of cellular dysfunction and cell death. Although restoration of blood flow is essential for recovery, reperfusion itself causes further damage, leading to a process of acute, sterile inflammation (51). Not being exclusive to transplantation medicine, damage after ischemia occurs in any tissue including the heart muscle following myocardial infarction or the brain after a stroke (52). In lung transplantation, this multi-factorial process leads to a complex pathology involving complex and broad molecular and cellular mechanisms (53). IRI thus distinguishes two phases of organ damage initiated with the discontinuation of blood supply (clamping of the organ during procurement) and a second phase at the time when blood flow is restored (reperfusion phase).

Reactive Oxygen Species (ROS) including superoxide, hydroxyl radicals and hydrogen peroxide play a key role in the development of IRI (54–57). Although low levels of ROS are a critical component of physiologic signaling pathways, the overload through both ischemia itself and reperfusion disturbs cellular function (58–60). This impairment is mainly driven by protein- and deoxyribonucleic acid-damage, alteration of signaling pathways and an augmentation of innate immune responses (61, 62). ROS are largely produced by alveolar type-II cells, vascular smooth muscle cells, endothelial cells and macrophages deriving from different sources including xanthine and NADPH oxidase, in addition to mitochondria (53, 63). Especially during reperfusion, restored oxygen facilitates the production of significant quantities of ROS (64, 65).

Of additional relevance, intracellular calcium overload has been proposed to be an initial step in the pathogenesis of the injury (66, 67). With a lack of oxygen, anaerobic glycolysis prevails, resulting in a decrease of intracellular pH caused by lactate and acid accumulation (65). Hydrogen ions accumulate leading to intracellular hypernatremia as a consequence of an accelerated Na^+^H^+^ exchange (68). Additionally, depletion of adenosine triphosphate (ATP) limits the activity of the Na^+^K^+^ ATPase and ATP-dependent calcium re-uptake. Hypernatremia leads to an additional calcium influx as accumulating Na^+^ is exchanged with Ca^2+^ (69). These mechanisms gain importance upon reperfusion, since the prompt normalization of the extracellular pH by pericellular washout results in a massive H^+^ gradient across the plasma membrane resulting in an accelerated calcium influx (65).

Both the excessive calcium accumulation and the overproduction of ROS represent key drivers for the formation of the mitochondrial permeability transition pore (mPTP) located at the inner mitochondrial membrane (70). Activation and opening of the mPTP has been proposed as one of the main driving forces of IRI (71–73). In addition to the structural damage caused by mitochondrial swelling, open mPTP facilitate the influx of hydrogen ions that uncouple the electron transport chain (ETC), further compromising ATP production (73, 74). Even under physiological conditions, ETC mediate a minor electron leakage. However, during reperfusion, electron leakage increases massively as a consequence of mitochondrial dysfunction with the production of large amounts of ROS once oxygen is reintroduced (75). Augmented ROS levels, damaged mitochondria with open mPTP accelerate ROS release furthermore, a process referred to as the ROS-induced ROS release (76, 77).

As part of the events during reperfusion, calcium overload and the excessive ROS generation trigger both, apoptosis and necrosis causing a further release of ROS, proinflammatory cytokines and damage-associated molecular patterns (DAMPs) consisting of peptides, proteins and nucleotide fragments (78). DAMPs represent endogenous danger signals that are normally carefully prevented from release to the extracellular space and differ from microorganism derived pathogen associated molecular patterns (PAMPs) (79, 80). Both PAMPs and DAMPs are mainly recognized by their pattern recognition receptors including Toll­like receptors that play an important role in the induction of innate immune responses (81–83).

Subsequently, the inflammatory cascade is initiated through, ROS- and DAMP-triggered activated resident lung macrophages as a key early source of multiple proinflammatory mediators that orchestrate lung IRI (84). This inflammatory milieu leads to pulmonary neutrophil infiltration, further exacerbating and maintaining lung inflammation and injury (85, 86). Released ROS and inflammatory cytokines upregulate and activate adhesion molecules including ICAM-1, CD18 and P-selectin on leukocytes and endothelial cells (79, 87). The activation of adhesion molecules facilitates the migration of neutrophils from their intravascular location to the lung interstitium where they release more ROS and proteolytic enzymes resulting into the destruction of cellular and extracellular matrix (88). This process is reinforced by an augmented expression of vascular endothelial growth factor during the hypoxic phase that increases vascular permeability during acute lung injury (89, 90). Complement activation seems to play an additional important role during IRI by mediating leukocyte chemotaxis and initiating cellular damage (91, 92). Moreover, widely injured endothelial cells decrease the production of nitric oxide facilitating, under physiological conditions, vasorelaxation, bronchodilation, immunomodulation, and maintenance of microvascular function (88, 93, 94). Additionally, neutrophil extracellular traps (NETs) have been found to accumulate in both, IRI and PGD after lung transplantation (95–97). Those data have also been confirmed clinically with higher concentrations of NETs in the bronchoalveolar lavage fluid of lung transplant recipients with PGD (96).

**Figure 1:**
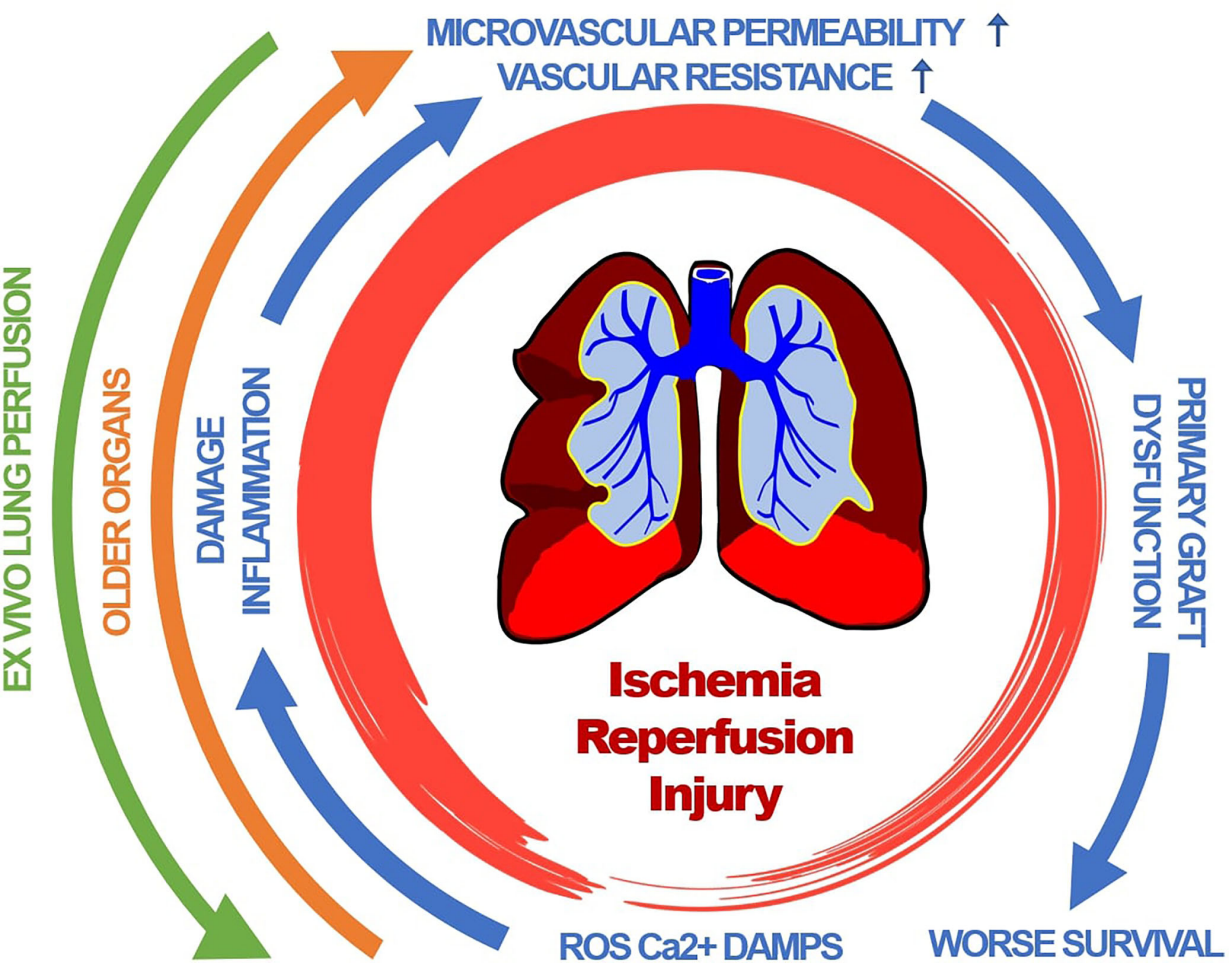
Primary graft dysfunction of lung transplants is driven by a complex cascade of pathophysiological events. Clinical hallmarks include pulmonary edema and compromised gas exchanges. Organ age accelerates those events. Therapeutic interventions include *ex-vivo* lung perfusion. ROS, reactive oxygen species; Ca2+, Calcium; DAMP, damage associated molecular pattern.

All these inflammatory pathways and mechanism contribute to an increased pulmonary vascular resistance and microvascular permeability (53, 98) leading to pulmonary edema and compromised gas exchanges as the clinical hallmarks of primary graft dysfunction of the lung (**Figure 1**) (29, 53).

## Impact of Aging on Ischemia Reperfusion Injury

Allogeneic lung transplantation represents the only curative approach for selected patients with end-stage lung disease. Advances in surgical techniques and immunosuppression therapy have improved graft survival rates to a median of 6.7 years compared to 4.7 year two decades ago (99). Of relevance, adult recipients who survived the first year after transplantation had a median survival of 8.9 years, emphasizing on the clinical relevance of PGD (100). Notably, with an increased experience and an augmented demand, donor age has increased steadily (101). Although early studies suggested that prolonged ischemia and increased donor age act synergistically towards worse survival rates, a recent report has failed to confirm this association (102–104).

While conclusive data in lung transplantation are currently lacking, data from other organ transplants support the concept that organ age augments damages subsequent to IRI: in hearts, this process has been driven, at least in part, through alterations in gene expression, signal transduction cascades, and mitochondrial dysfunction, resulting in an impaired intrinsic tolerance against damaging stress (105, 106). Notably, a decline of silent information regulator of transcription 3 (SIRT3) protein with age appears to be associated with an augmented damage in older hearts (107). There is also other strong experimental evidence that aging enhances the susceptibility of IRI in liver transplantation (108). A multivariate analysis of potential risk factors in orthotopic liver transplantation revealed that both donor age and prolonged cold ischemia times were independently associated with a higher incidence of primary dysfunction (109, 110). Experimentally, older livers have been more susceptible to IRI, linked to a depletion of both sirtuin-1 and mitofusin-2, resulting in mPTP onset/mitochondrial dysfunction, and cell death (111). Of additional interest, pretreating old rats with pooled young plasma appeared to reduce age-dependent liver IRI (112). It was further demonstrated that old rats experience more severe consequences of kidney IRI linked to an augmented immune response and increased oxidative stress, mechanisms that may also apply for IRI in lung transplantation (113, 114). A synergistic relationship between donor age and prolonged ischemia was also shown in an experimental renal allograft model leading to both functional and morphological deterioration after transplantation (115, 116).

Aging also impacts graft immunogenicity. In a broad clinical analysis of renal transplant recipients listed in the UNOS data base, we have been able to show that older grafts had higher rates of acute rejection within the first post-transplant year. Conversely, acute rejections were significantly lower in older recipients although they were more likely to receive an older organ (117). That grafts from older organs are more immunogenic has also been confirmed experimentally with an increase in T-cell alloreactivity, cytokine production observed early after transplantation (118). Moreover, it has also been shown that older donor age and prolonged warm ischemia time are both associated with an increased risk for rejection, contributing synergistically towards an augmented innate immune activation (119).

Clinically, elevated perioperative levels of cell-free circulating plasma mitochondrial DNA (cf-mt-DNA) have been observed in lung transplant recipients with moderate or severe PGD (120). We have recently shown that organ age and IRI act synergistically, leading to an increased DAMP release. Old mice that underwent renal IRI showed a 15x increase in cf-mt-DNA levels that act as a DAMP, inducing sterile inflammation. Remarkably, the pretreatment with senolytics that selectively kill senescent cells prior to IRI had the capacity to reduce both cf-mt-DNA and pro-inflammatory T-cells. These senescent cells accumulate with age and contribute through the secretion of a myriad of pro-inflammatory factors termed the senescence-associated secretory phenotype (SASP) to a proinflammatory environment (121–124). Of additional clinical relevance, senolytics applied to organ donors prior to procurement, prolonged the survival of old cardiac allografts beyond that of young donors (125). Most recently, experimental models have indicated that IRI itself can induce senescence and that senolytics have the potential to ameliorate this injury (126–128). Notably, senolytics have been tested before in patients with idiopathic pulmonary fibrosis. This first in-human study supported the feasibility of senolytics to interfere with lung injury (129, 130). Thus, senolytics may represent, at least in theory, a therapeutic opportunity to improve the equality of older lungs for transplantation.

## *Ex vivo* Lung Perfusion

Due to peri transplant complications such as PGD and its impact on long-term survival, careful selection of donor lungs is crucial, however, these can lead to lower donor utilization rates of 15% to 25% from multiorgan donors (131–133). Mortality remains high for patients waiting for lung transplantation. In 2017, 326 patients died or became too sick to undergo lung transplantation in the US accounting for > than 10% of the removals (134). In the past decade, modern technologies including *Ex vivo* lung perfusion (EVLP) have facilitated the evaluation and reconditioning of marginal donor lungs including those from older lungs, thus augmenting the pool of prospective donor organs (135, 136). Notably, lungs from older donors are more likely to undergo EVLP (137). This novel approach allows explanted donor lungs to be preserved in perfused, ventilated and normothermic condition, decreasing tissue ischemia and lung damage (138). Three different systems and protocols are currently used clinically: the Vivoline^®^ LS1 system (Vivoline Medical, Lund, Sweden), the Organ Care System™ Lung (OCS, Transmedics, Andover, MA), and the XPS™ XVIVO Perfusion AB system (XVIVO Perfusion, Goetheborg, Sweden) (139).

In many ways, lung preservation has ploughed the way for novel preservation methods for other organs. In a prospective, non-randomized clinical trial, the Toronto group transplanted 20 high risk lungs which were evaluated for 4 hours while being perfused *ex-vivo* by the XVIVO Perfusion AB system. During this two-year period, 116 non-high-risk lungs were transplanted without EVLP and used as controls. Lungs in the EVLP group showed an improved PaO_2_/FiO_2_ ratio after EVLP resulting into a reduced PGD incidence (140). Recently, a randomized, open-label, phase 3 trial used the OCS system in 151 patients and compared them to 169 standard protocol recipients (cold static storage). This study also demonstrated reduced PGD rates (with and without EVLP: vs 29.7% vs. 17.7%, p=0.015), however not resulting into improved short-term survival (141).

## Conclusion

Understanding the link between organ age, IRI and graft immunogenicity will be critical in optimally utilizing available lungs for transplantation. Although, the bulk of available data both, clinically and experimentally is currently provided through evidence outside of lung transplantation it can be assumed that data from other organ systems do also apply for lungs. The elimination of senescent cells and the assessment of older organs on machine perfusion devices may help safely increase the number of available lungs for transplant.

## Author Contributions

All authors listed have made substantial contributions to the concept of this work, helped writing and editing the manuscript and approved the final version for publication.

## Funding

This work was supported by the Biomedical Education Program (BMEP) of the German Academic Exchange Service (MJR and AS), the Osaka Medical College Foundation (to TM), NIH grant R01AG064165 (to SGT) and a grant by the Pepper Foundation (to HZ).

## Conflict of Interest

The authors declare that the research was conducted in the absence of any commercial or financial relationships that could be construed as a potential conflict of interest.

## Publisher’s Note

All claims expressed in this article are solely those of the authors and do not necessarily represent those of their affiliated organizations, or those of the publisher, the editors and the reviewers. Any product that may be evaluated in this article, or claim that may be made by its manufacturer, is not guaranteed or endorsed by the publisher.
